# Surface and Bulk Carbide Transformations in High-Speed Steel

**DOI:** 10.1038/srep16202

**Published:** 2015-11-05

**Authors:** M. Godec, T. Večko Pirtovšek, B. Šetina Batič, P. McGuiness, J. Burja, B. Podgornik

**Affiliations:** 1 Institute of Metals and Technology, Lepi pot 11, 1000 Ljubljana, Slovenia; 2 Metal Ravne d.o.o., Koroška cesta 14, 2390 Ravne na Koroškem, Slovenia.

## Abstract

We have studied the transformation of carbides in AISI M42 high-speed steels in the temperature window used for forging. The annealing was found to result in the partial transformation of the large, metastable M_2_C carbides into small, more stable grains of M_6_C, with an associated change in the crystal orientation. In addition, MC carbides form during the transformation of M_2_C to M_6_C. From the high-speed-steel production point of view, it is beneficial to have large, metastable carbides in the cast structure, which later during annealing, before the forging, transform into a structure of polycrystalline carbides. Such carbides can be easily decomposed into several small carbides, which are then randomly distributed in the microstructure. The results also show an interesting difference in the carbide-transformation reactions on the surface versus the bulk of the alloy, which has implications for *in-situ* studies of bulk phenomena that are based on surface observations.

High-speed steel (HSS), which has been in use for almost 100 years, remains as one of the most important of today’s tool materials and is employed in the manufacture of taps, dies, drills, saw blades and other cutting equipment. The name “high-speed steel” derives from the fact that tools made from this material are capable of cutting materials at much higher speeds than conventional carbon tool steel, retaining their hardness and ability to cut even when the tip of the tool reaches low red heat^1^. In addition to carbon, high-speed steels are usually alloyed with tungsten, molybdenum, vanadium and cobalt, all of which are known for their ability to form carbides. In fact, it is these carbides that give high-speed steels their high hardness and excellent wear resistance. However, these useful properties of high-speed steels are very much dependent on the type, size, shape, amount and distribution of the carbides^2^,^3^,^4^,^5^,^6^,^7^. As a consequence, a detailed investigation and evaluation of the carbides that form in high-speed steel is crucial.

The AISI M42 super-high-speed steel is characterized by its high molybdenum content and belongs to the ledeburitic group of steels, because the last transformation during solidification is eutectic. The cast microstructure of these ledeburitic steels generally consists of dendrites surrounded by a more-or-less connected network of eutectic carbides^8^; however, due to the wide solidification range and the complex eutectic reaction it tends to segregate and form different types of carbides: either M_2_C or M_6_C in the case of conventional high-speed steels. The final microstructure of the steel consists of ferrite with fine carbide precipitates and ledeburite with primary carbides in between^9^.

During conventional tool-steel production, the steel is cast and then forged in the ideal temperature window of 1100–1200 °C. At these temperatures the metastable M_2_C carbide phase transforms into other, more stable, carbides. The reports in the literature indicate that the M_2_C carbides transform to M_6_C and MC carbides following the path M_2_C + matrix → M_6_C + MC^2^,^3^,^10^,^11^,^12^,^13^,^14^. These primary and eutectic carbides can then be broken down into smaller carbides as a result of hot deformation (e.g., forging) or mechanical fragmentation. Another possibility is to anneal the steel, so that the exchange of elements is driven by diffusion processes. During annealing, the primary and eutectic carbides decompose, which means the compositions of the carbides change. Furthermore, when held at an elevated temperature for sufficient time, all types of carbides can agglomerate and spheroidize in order to decrease their surface energy and achieve a more stable state^13^. Thus, it is beneficial to optimize the heat treatment prior to forging in order to obtain the desired size and distribution of the carbides.

Electron-backscatter diffraction (EBSD) is an excellent technique for characterising microstructures and analysing carbide phases^4^,^15^,^16^,^17^,^18^. In recent years, *in-situ* EBSD analysis has attracted researchers wishing to study the deformation of HSS^19^ as well as grain growth and phase transformation by employing a heating stage^20^,^21^,^22^. It is also possible to conduct a form of quasi-*in-situ* EBSD by marking the area of interest on the sample, annealing/heating the sample, and then replacing it in the microscope and repeating the EBSD analysis at the same location on the sample^23^. In this way, changes to the microstructure, especially the eutectic carbides, caused by the annealing, can be investigated and analysed.

The aim of our work was to investigate the cast microstructure of the super-high-speed steel AISI M42 and to examine the changes to the eutectic carbides during annealing at 1100 °C for various times. We wanted to test the hypothesis that the processes we observe on the surface are the same as the processes taking place in the bulk of the material. In order to do this we performed quasi-*in-situ* studies of the carbide transformations on the surface and in the bulk. We also wanted to determine whether the different conditions that necessarily occur at a free surface, compared to the bulk, during a heat treatment can prevent us from obtaining truly representative results.

## Experimental Details

### Material and specimen preparation

The composition of the AISI M42 molybdenum super-high-speed steel is listed in Table 1. This steel was delivered in the as-cast condition, without any additional electro slag remelting. After casting, the steel ingot was cut in half, with the specimens being taken from the middle section. Because the main goal of the experiment was to observe the transformation of the carbides on the free surface and in the bulk during annealing, two sets of specimens were prepared. For the observation of the processes on the free surface, a specimen was prepared for EBSD analysis—with the final steps being diamond polishing and OPS (silica oxide colloidal suspension of 40 nm size) polishing for 5 minutes—prior to annealing. In this way, the specimens could be observed immediately after annealing, and no additional surface preparation was necessary. For the observation of the processes in the bulk, the specimens were first annealed and then cut in half and prepared for an optimal EBDS analysis (ground, polished, OPS).

### Annealing

The specimens were annealed in a vacuum system using a stub-like heating stage in an argon atmosphere at 100 mbar to ensure an optimally homogeneous heat distribution in the specimens. The specimens were first stabilized in the vacuum system at 400 °C, followed by high-temperature annealing, which was performed at 1100 °C and 1150 °C for 15 to 60 minutes, in order to follow the transformation of the carbides. The temperature was monitored at all times using a thermocouple mounted on the surface of the specimen. After the annealing step, the specimens were left to cool in the vacuum chamber in the Ar atmosphere. Due to the small size of the specimens and the presence of Ar in the vacuum chamber, the cooling was quite rapid at around 10 K/s, which effectively froze the microstructure. The residual contents of oxygen and carbon in the chamber were negligible, so no oxidation or carbon contamination was expected. In our previous experiments^24^ a similar heating system and heating conditions were employed. However, subsequent detailed analyses revealed that local short circuits caused material melting, leading to an incorrect interpretation of the data. This melting results in the deposition of material on the surface and in a different surface-carbide transformation than would be expected in totally clean conditions. Here we must stress that the carbide transformation is extremely dependent on the quality of the vacuum. Therefore, a heating-system modification involving high-temperature annealing in argon gas was introduced.

### Analysis

The specimens in the first set, which were intended for an analysis of the carbides in the bulk, were prepared as described above to ensure good-quality diffraction patterns. The specimens prepared for the observation of carbide transformations on the free surface were immediately analysed after annealing with no subsequent surface preparation. Due to the relatively large size of the carbides, the area of interest on the specimen surface could be marked, and the same area could be subsequently analysed after different annealing times.

The imaging and EBSD analyses were performed using a FEG SEM JEOL 6500F field-emission scanning electron microscope with energy-dispersive spectroscopy (an INCA X-SIGHT LH2-type detector, INCA ENERGY 450 software) and EBSD (HKL Nordlys II EBSD camera using Channel 5 software). The instrument was operated at 15 kV and a 1.3-nA current for the EBSD analysis, with a tilting angle of 72 degrees. Individual diffraction patterns were obtained, as well as mapping of the areas of interest. The detection was set to 5–7 bands, 4 × 4 binning. The typical step size was 0.5 μm and the indexing rate was between 95% and 97%. Minimal post-processing was performed in the case of the mappings, which was limited to removing the so-called “wild spikes”. A summary of the EBSD “match units” for the individual carbides is given in Table 2. It is important to point out that the EBSD determination of the carbides is based on their crystallography and not necessarily on their composition. Thus, the Fe_3_W_3_C “match unit” is a representative of the M_6_C carbide, but, for example, Mo, or other elements, could also be present in this carbide type.

## Results and Discussion

### Starting material

**Figure 1:**
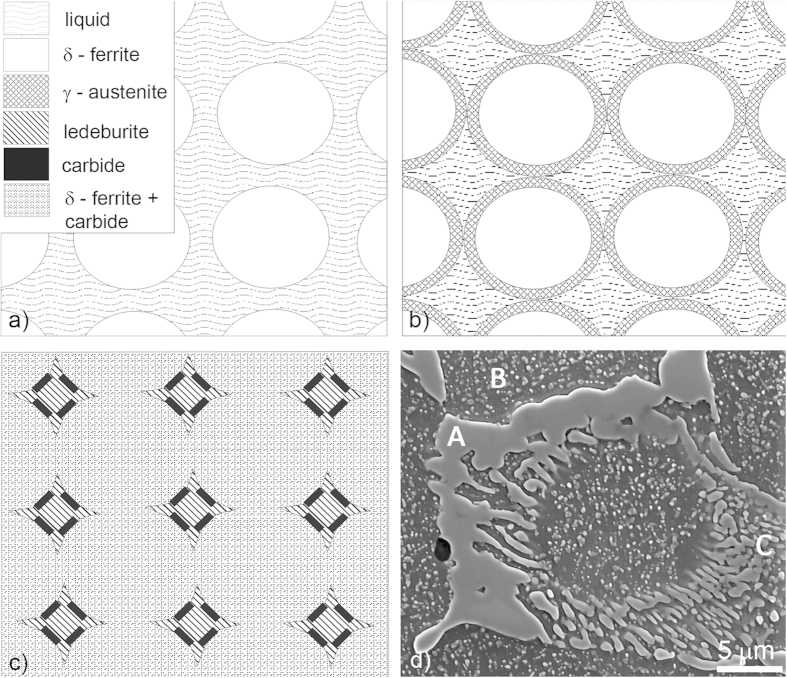
Sketches of the microstructures during the solidification process with the microstructure legend (**a**) microstructure at approximately 1300 °C, (**b**) microstructure at approximately 1250 °C, (**c**) microstructure at room temperature, (**d**) SE image of the microstructure with marked phases: A—M_2_C or M_6_C primary carbides, B—ferrite and carbides precipitates, C—ledeburite (ferrite and M_2_C or M_6_C carbides).

The AISI M42 steel has a ledeburitic microstructure. It consists of a dendritic matrix of α-ferrite and smaller carbides, mostly M_6_C, M_7_C_3_, M_2_C, large primary M_2_C carbides and a ledeburite of carbides and ferrite. This microstructure develops during the solidification process, which is sketched in Fig. 1. First, a skeleton of the primary delta-ferrite dendrites is formed, with a progressive enrichment of the remaining adjacent liquid^8^. As the temperature is reduced, a peritectic reaction occurs and consumes the ferrite, forming a rim of austenite around the delta ferrite and the primary carbides. The ledeburitic eutectic is a fine-grained mixture of eutectic carbides and austenite nucleates, and grows in the remaining liquid phase in the interdendritic spaces. The final cast microstructure of AISI M42 ledeburitic steel is compounded by dendrites surrounded by a more-or-less connected net of eutectic carbides. Depending on the chemical composition of the M42 steel and the solidification conditions, either the eutectic metastable M_2_C or the stable M_6_C phase forms^8^,^25^.

**Figure 2:**
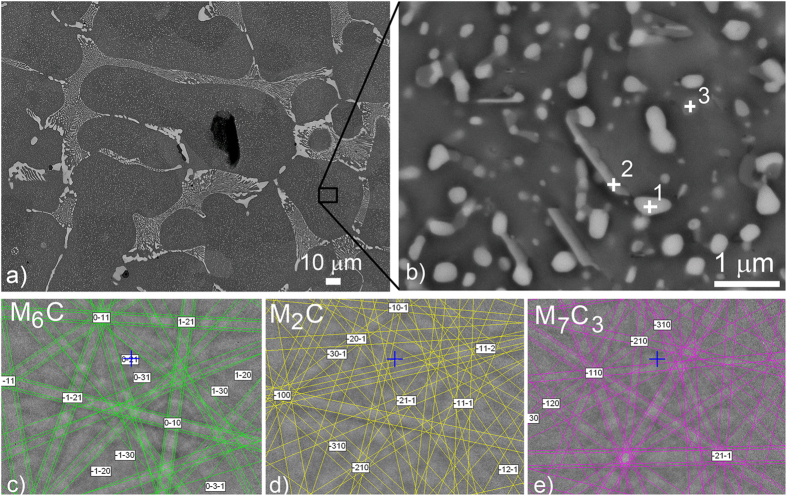
(**a**) BE image of the as-received specimen of AISI M42 steel without any annealing (**b**) BE close-up image of matrix, which consists of ferrite and different carbides. In the image the spots of the EBSD phase analysis are marked. (**c**) Solved Kikuchi pattern obtained at spot 1. (**d**) Solved Kikuchi pattern obtained at spot 2. (**e**) Solved Kikuchi pattern obtained at spot 3.

A backscattered-electron (BE) image of the microstructure of the specimen is shown in Fig. 2(a), where all the phases in the microstructure can be seen. A large number of small carbides are present in the ferrite phase^26^. Figure 2(b) is a close-up of the ferrite phase, where the smaller carbides can be seen. Due to the small size of these carbides and their overlapping Kikuchi patterns, it is rather difficult to identify them. However, three different types of small carbides were successfully indexed in the matrix. The brighter, spherical carbides were determined to be M_6_C carbides (Fig. 2c), the needle-like carbides were successfully identified as M_2_C carbides (Fig. 2d), and the darker, spherical carbides were determined to be M_7_C_3_-type carbides (Fig. 2e). Most of the carbides are bound together and form clusters of two or more different types of carbides. Carbides smaller than 100 nm cannot be accurately analysed by EBSD due to the presence of overlapping patterns. Hetzner and Van Geertruyden^26^ reported that the AISI M62 high-speed-steel matrix consists of martensite and a large volume fraction of carbides with two morphologies, i.e., rods and globular. In their study, the rods were identified as M_2_C carbides, and the globular-shaped carbides were determined to be M_6_C carbides. This is consistent with our experiment, where the needle-shaped carbides were also determined to be M_2_C carbides with an orthorhombic symmetry, which is reflected in their form.

**Figure 3:**
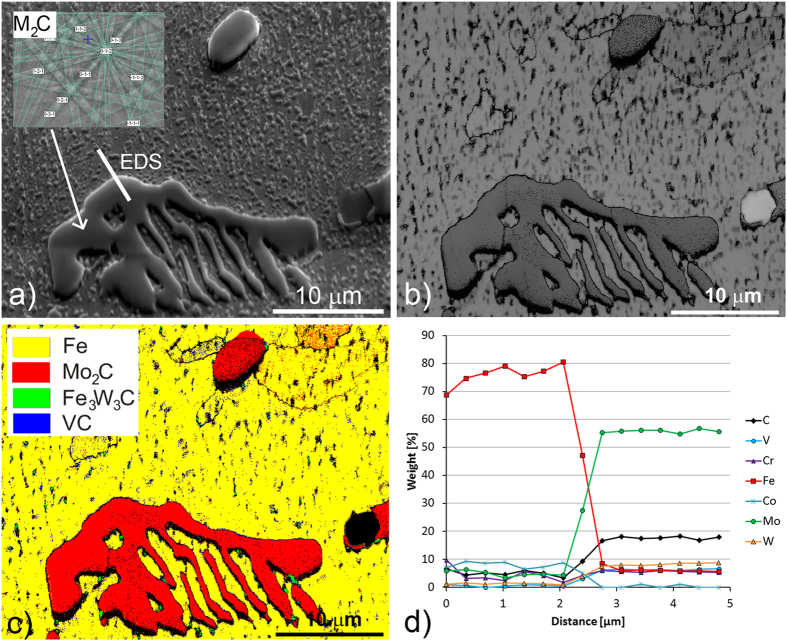
Microstructure of as-cast AISI M42 steel, (**a**) secondary-electron image, (**b**) EBSD band-contrast image, (**c**) EBSD phase map with phase-colour legend, (**d**) EDS spot analysis along the line marked in (**a**).

Figure 3 shows a typical example of a eutectic carbide that is present in the steel. These carbides are in the form of M_2_C, with a small amount of the M_6_C-type carbides, most likely not eutectic. The presence of MC carbides is not confirmed, blue spots in Fig. 3(c) are too small to be indexed in the EBSD mapping, which means that the carbides are not present or too small for analysis. The carbide dendritic shape, a result of the EBDS spot analysis, is a resolved Kikuchi pattern representing the M_2_C-type carbide, Fig. 3(a). The EBSD band-contrast image (Fig. 3b) reveals the details in the microstructure, especially the grain boundaries. From this it is clear that the M_2_C carbide is monocrystalline, as no grain boundaries can be observed. The EBSD phase-map analysis, shown in Fig. 3(c), supports the idea that the primary and eutectic carbides are homogeneous M_2_C. Although it is rather difficult to distinguish any chemical inhomogeneity using the energy-dispersive spectroscopy (EDS) technique due to the large analysis volume, the EDS spot line analysis performed across the matrix-carbide boundary indicates a homogeneous composition throughout the carbide phase. As shown in Fig. 3d, the analysed M_2_C carbide contains mainly Mo, with minor amounts of W, V, Cr, and Fe atoms.

### Carbide transformation in the bulk during annealing

**Figure 4:**
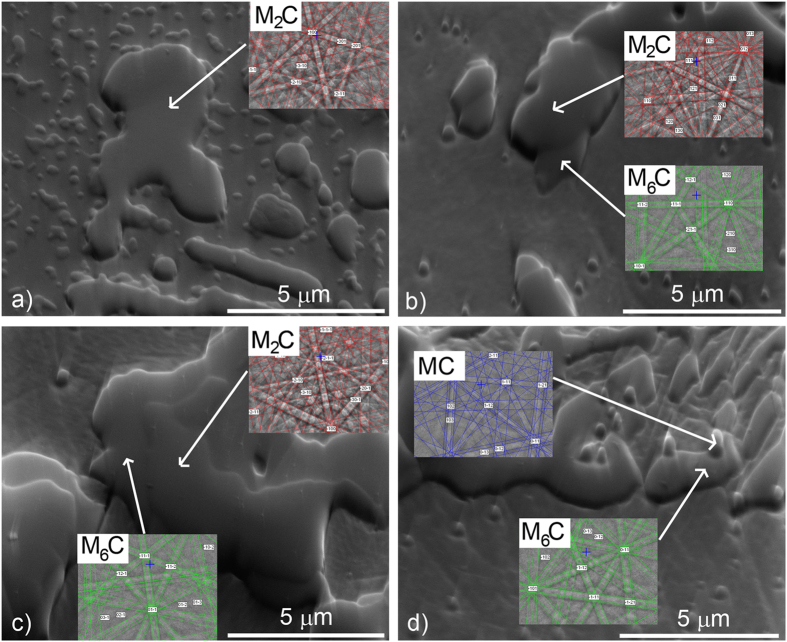
Microstructure changes during annealing at 1100 °C for different times followed by EBSD point analysis (secondary-electron images); (**a**) as cast, (**b**) 15-minutes annealing, (**c**) 30-minutes annealing, (**d**) 60-minutes annealing.

In our research the SEM-based EBSD technique was applied to track the carbide transformation during the annealing at 1100 °C ad 1150 °C for different annealing times. The microstructural changes taking place during the annealing are shown in Fig. 4. The as-cast steel contains metastable M_2_C carbides (Fig. 4a), which transform mostly into M_6_C carbides and a very small amount of MC during the high-temperature annealing. In the case of AISI M42 the amount of MC depends on the vanadium concentration. The evidence from the EBSD mapping shows that the transformation starts on the boundary between the carbide and the ferrite, and then grows towards the inner region. Due to low vanadium concentration and consequent most probably small amount of MC, during annealing for 15 minutes at 1100 °C the expected transformation: M_2_C + matrix → M_6_C + MC + matrix was not observed. A large specimen area was observed and no MC carbides were found in the EBSD maps or observed as hills in the tilted secondary-electron (SE) image. However, we did observe the M_6_C carbides. A longer annealing time (30 minutes) caused almost half of the metastable M_2_C carbides to transform to M_6_C carbides, which grew from all directions towards the centre of the M_2_C carbide. The previously monocrystalline M_2_C carbide transforms to polycrystalline M_6_C carbides with different orientations (Fig. 4c). Again, no MC carbides were observed in the microstructure. However, after annealing for an hour at the same temperature, the MC carbides were confirmed in the microstructure. In addition most of MC carbides were observed in the ledeburitic areas at the M_6_C grain boundaries (Fig. 4d).

**Figure 5:**
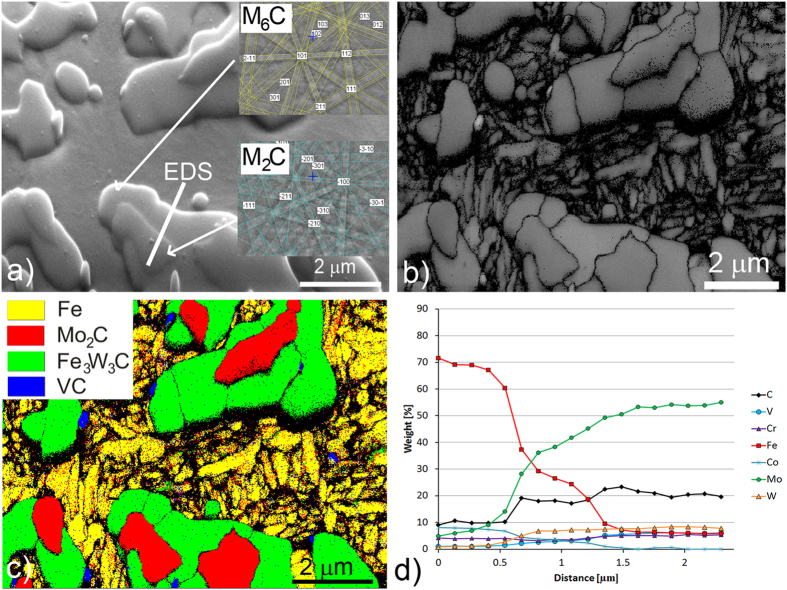
Microstructure development during annealing at 1100 °C for 60 minutes in ledeburitic phase; (**a**) secondary-electron image with EBSD spot analysis and marked line of EDS analysis (**b**) EBSD band-contrast image, (**c**) EBSD phase map with phase-colour legend, (**d**) EDS spot analysis in line marked in (**a**).

The results of the EBSD phase-map analysis of the specimen annealed at 1100 °C for 60 minutes are shown in Fig. 5. It appears that during annealing at 1100 °C for 60 minutes part of the M_2_C carbides transformed into the M_6_C carbide type, with some additional MC carbides (Fig. 5c). The EDS analyses of the individual carbides showed that the M_6_C carbides were rich in both iron and molybdenum, while the M_2_C carbides were mostly molybdenum. However, some other carbide-forming elements can be exchanged in the crystal structure. The EDS line profile across the M_6_C carbide growth boundary, as shown in Fig. 5d, clearly indicates that the transformation of the carbide is driven by the atomic diffusion of molybdenum and iron. The M_6_C carbide transformation starts on the carbide surface, after which the phase boundary moves towards the centre of the carbide. At the same time, the crystal structure transforms from orthorhombic M_2_C to cubic M_6_C. However, as long as the concentration of elements remains below a certain level, the crystal transformation cannot occur. A comparison of the EBSD band-contrast and phase maps for the as-cast and annealed specimens reveals a change in microstructure occurring during the annealing (Fig. 5b). During annealing, the MC carbides form alongside the transformation of the M_2_C carbides into M_6_C, with the large M_2_C carbides transforming mainly into a few, small-grained M_6_C carbides with a different crystal orientation. This phenomenon has a beneficial effect on the final microstructure of forged-and-annealed AISI M42 steel. During production, the AISI M42 high-speed steel goes through a plastic deformation, which is the result of the forging process at high temperature. During this plastic deformation the eutectic carbides transform to a more stable form and break into smaller and more uniformly shaped carbides^24^,^25^. Because the large monocrystal M_2_C carbides transform into differently oriented M_6_C carbides, they can be broken down more easily during forging. Due to the carbide transformation, the final microstructure of the forged-and-annealed AISI M42 steel has smaller M_6_C and MC carbides, the same as if the starting material already contained stable M_6_C carbides.

Reports in the literature suggest that, in general, the M_2_C carbide transformation follows the path: M_2_C + matrix → M_6_C + MC + matrix^2^,^3^,^10^,^11^,^12^,^13^,^14^. Figure 5d shows that the M_2_C carbide is richer in V than the M_6_C carbide; therefore, it can be concluded that the content of vanadium controls the amount of MC carbides formed. The vanadium has to diffuse out of M_2_C carbides in order to make way for the newly formed M_6_C carbides that have decreased solubility of vanadium. This might provide an explanation as to why the MC carbides are mostly formed at the M_6_C carbide grain boundaries.

To better understand the process of carbide transformation, let us take a look at what is happening during the solidification. Based on our observations, we propose the following transformation. The metastable, primarily M_2_C monocrystalline carbide plus the matrix transform into stable polycrystalline M_6_C carbides. The metastable ledeburitic M_2_C monocrystalline carbide plus the matrix transform into stable polycrystalline M_6_C and monocrystalline MC carbides. At higher temperatures, due to an increased vanadium concentration more MC carbides form in the ledeburitic area. It is obvious that during the solidification process a higher concentration of vanadium is present in the last solidified region, which is the ledeburitic area.

**Figure 6:**
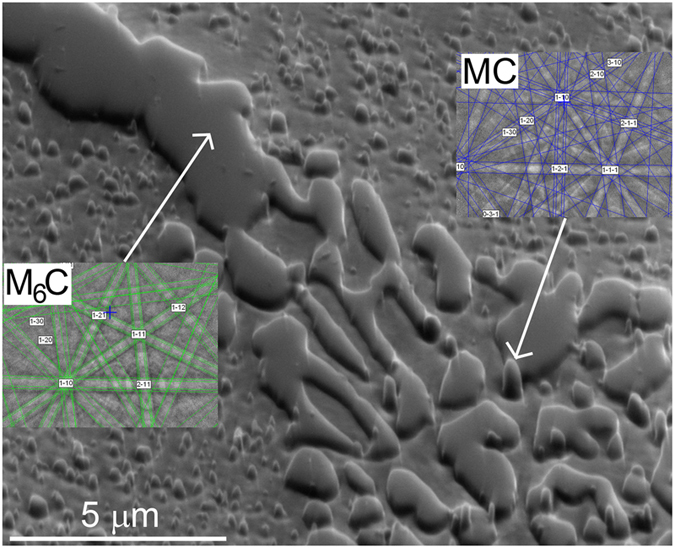
Microstructure development during annealing at 1150 °C for 60 minutes. All M_2_C carbides have transformed into M_6_C carbides and the MC carbide precipitates are mainly observed in the ledeburitic phase.

Figure 6 shows ledeburite in the centre image and a larger primary carbide on the top-right of the ledeburite. All the M_2_C carbides, both primary and those from the ledeburite, are transformed into the M_6_C carbide. In the ledeburite region the MC carbides are easily observed. This is typical for most of the observed microstructure. The other area where the MC carbides form is on the phase boundary of the M_6_C carbides transformed from the primary M_2_C carbide.

### Carbide transformation during annealing on the free surface

**Figure 7:**
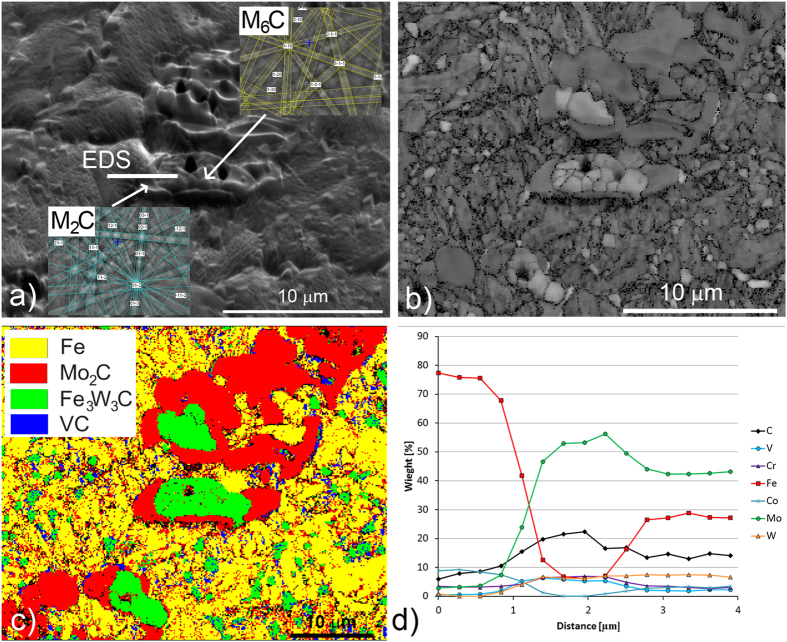
Microstructure development during annealing at 1100 °C for 30 minutes on the surface; (**a**) secondary-electron image with EBSD spot analysis and marked line of EDS analysis (**b**) EBSD band-contrast image, (**c**) EBSD phase map with phase-colour legend, (**d**) EDS spot analysis in line marked in (**a**).

From the *in-situ*-analysis point of view it is very important to identify what is happening with the microstructure on the free surface and compare it with the process in the bulk. Figure 7 shows the microstructure development of the free surface during annealing at 1100 °C for 30 minutes in a protective atmosphere of argon. As is clear from the image, this process is not the same as that occurring in the bulk.

In comparison with the processes in the bulk, the surface is slightly different: many M_6_C carbides were found on the top of the M_2_C carbides and in the matrix, and small MC carbides are also present on the surface of the annealed specimen. Most probably, the M_6_C carbides start to form on the surface in the matrix-M_2_C phase boundary. The EDS spot line analysis showed a similar composition for the M_6_C carbides formed in the bulk (Fig. 5d) and on the *in-situ*-annealed surface specimens (Fig. 7d), while the M_2_C carbides had a higher concentration of Fe atoms in the case of the *in-situ*-annealed surface specimens.

In an earlier investigation^24^ we looked at a different annealing set-up: the specimens were kept in a vacuum on Mo boats and were heated by a large direct current sent through the specimens. However, we discovered later that this set-up could introduce some Mo contamination on the surface of the specimens due to evaporation from the Mo boat. For this reason we introduced a completely different vacuum-annealing procedure using a stub heater in a protective atmosphere of Ar to ensure that there was no contamination from the heating element.

## Discussion

### Mechanism of carbide transformation in the bulk and on the free surface

**Figure 8:**
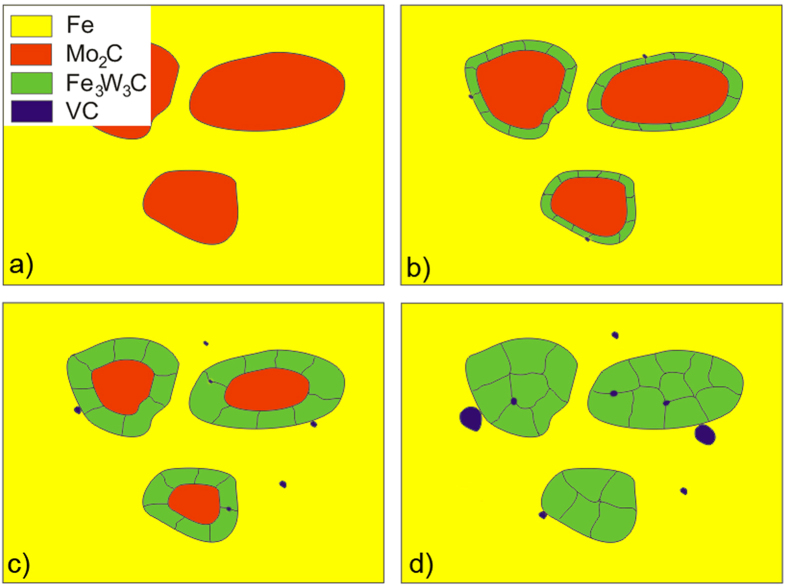
Sketch of microstructure development in bulk during specimen annealing; (**a**) as-cast starting microstructure (**b**) annealed at 1100 °C for 15–30 minutes, (**c**) annealed at 1100 °C for 60 minutes, (**d**) annealed at 1150 °C for 60 minutes.

**Figure 9:**
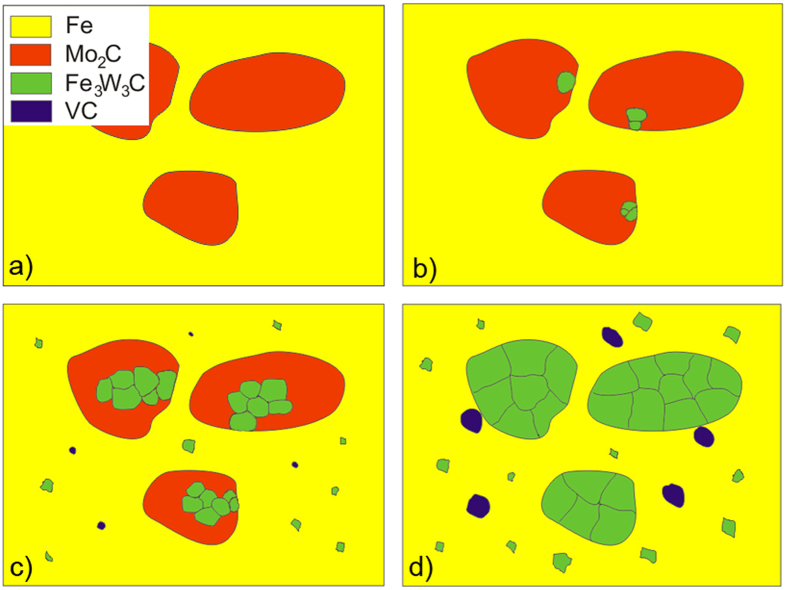
Sketch of microstructure development on the surface during specimen annealing; (**a**) as-cast starting microstructure (**b**) annealed at 1100 °C for 15–30 minutes, (**c**) annealed at 1100 °C for 60 minutes, (**d**) annealed at 1150 °C for 60 minutes.

Figure 8 shows a sketch of the bulk-microstructure development process during annealing, while Fig. 9 is a sketch of the process for the surface-microstructure development during annealing. In both cases the starting microstructure consists of unstable M_2_C eutectic carbides in a ferrite matrix. In the case of a bulk transformation during the annealing, the unstable M_2_C carbides transform into small, stable M_6_C and MC carbides with a different crystal orientation.

The sketch of the surface-microstructure development shows a different route for the carbide transformation due to the effect of the free surface and, consequently, the different diffusion processes. High-temperature annealing leads to all of the M_2_C carbides being covered with a thin layer of M_6_C carbides. At higher temperatures the matrix surface becomes covered with M_6_C carbides.

This difference seems to be due to the migration of carbide forming atoms on the surface as a result of the surface diffusion, which is much faster than the bulk diffusion^27^, and is very likely the reason for the different carbide-transformation phenomena. *in-situ* and quasi-*in-situ* studies of the phase transformations might not always give results that are representative of the actual processes taking place in the bulk of the material, which could be attributed to completely different diffusion conditions in the bulk and on the free surface, but also the temperature variations within the specimen and the compositional inhomogeneity could play a role. Nonetheless, it is important to note that the results obtained when using the *in-situ* studies on surfaces need to be interpreted carefully when referring to the material properties.

## Conclusions

In summary, the microstructure development of the as-cast, molybdenum-containing, super-high-speed steel AISI M42 was studied in order to clarify the carbide-transformation phenomena and to show that *in-situ* observations do not always accurately represent the transformation phenomena taking place in the bulk of the material. An as-cast microstructure was chosen with unstable primary and eutectic M_2_C carbides in the ferrite matrix. Annealing resulted in a partial transformation of the large M_2_C carbides into several small and more stable grains of M_6_C and small MC carbides, with an associated change in the crystal orientation. This carbide transformation phenomenon has a beneficial effect during forging, and therefore it can contribute to the design of future thermo-mechanical processes for high-speed steels.

The results have also shown an interesting difference in the carbide-transformation reactions on the surface versus the bulk of the alloy, presumably due to the operation of different diffusion processes. An *in-situ* study of surface-driven phenomena during the annealing of AISI M42 super-high-speed steel was found to give different results compared to the bulk. In the case of the bulk, the M_2_C-to-M_6_C transformation starts at the M_2_C/matrix boundary and uniformly propagates inwards. However, on the free-surface, although starting at the M_2_C/matrix boundary, the transformation kinetics are non-uniform. Due to faster surface diffusion the atoms tend to migrate on top of the existing carbide and form a new, stable carbide. Similar processes can also be observed in the matrix, where the stable carbides precipitate and form a new carbide layer.

This study shows that special attention should be paid during the *in-situ* studies of diffusion-driven transformations where the phenomena observed on the free surface are not necessarily the same as those observed in the bulk. This was demonstrated in the case of carbide transformations, and it is reasonable to expect that there are other, similar examples.

## Additional Information

**How to cite this article**: Godec, M. *et al.* Surface and Bulk Carbide Transformations in High-Speed Steel. *Sci. Rep.* **5**, 16202; doi: 10.1038/srep16202 (2015).

## Figures and Tables

**Table 1:** Composition (wt%) of AISI M42 high speed steel used in the experiment.

C	Cr	Mo	W	V	Co	Fe
1.1%	3.8%	9.5%	1.5%	1.2%	8.0%	balance

**Table 2:** EBSD “match units”.

Carbide	Space group	Laue group
M_2_C (Mo_2_C)	60, Pbcn	3 mmm
M_6_C (Fe_3_W_3_C)	227, F d-3m	11 m3m
MC (VC)	225, F m-3m	11 m-3m
M_7_C_3_ (Cr_7_C_3_)*	62, P nma	3 mmm

^*^This was not used as a “match unit”.
